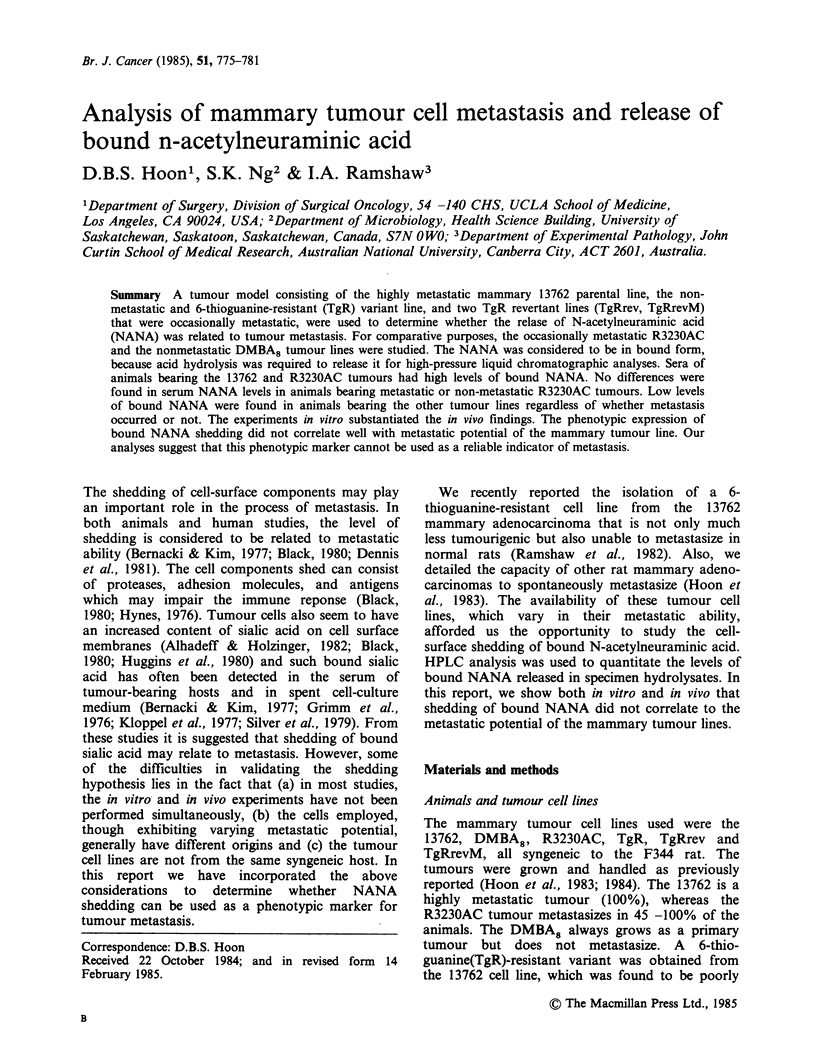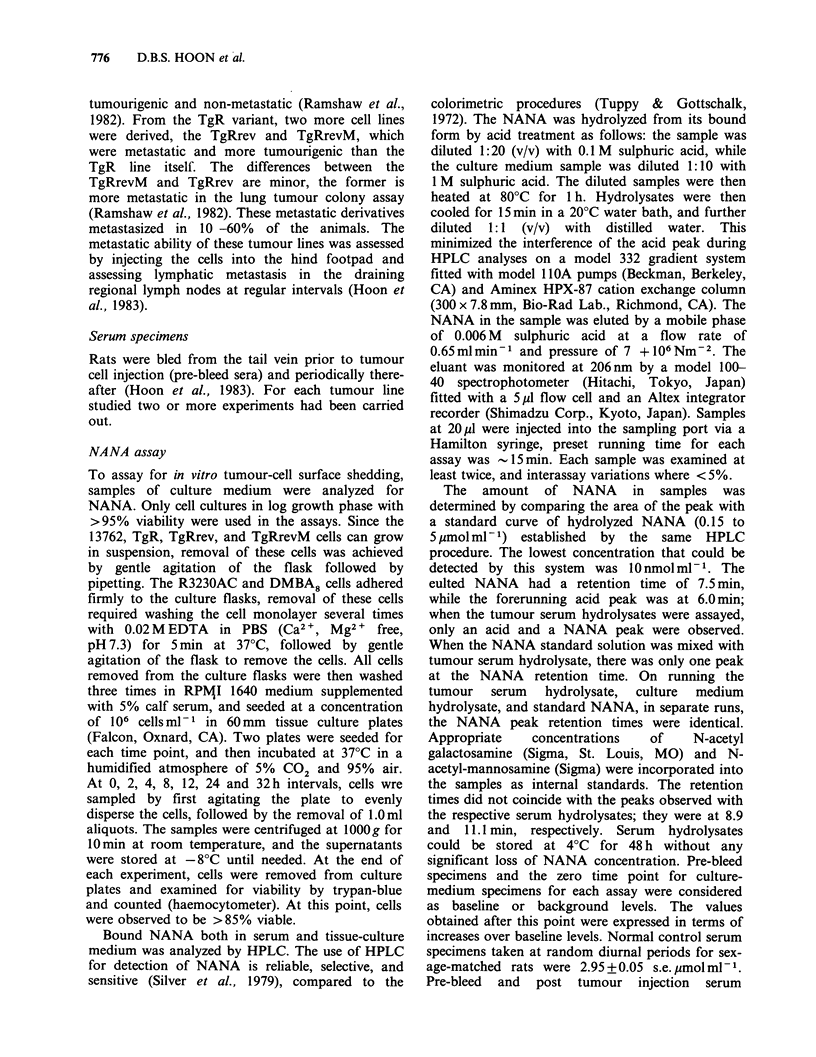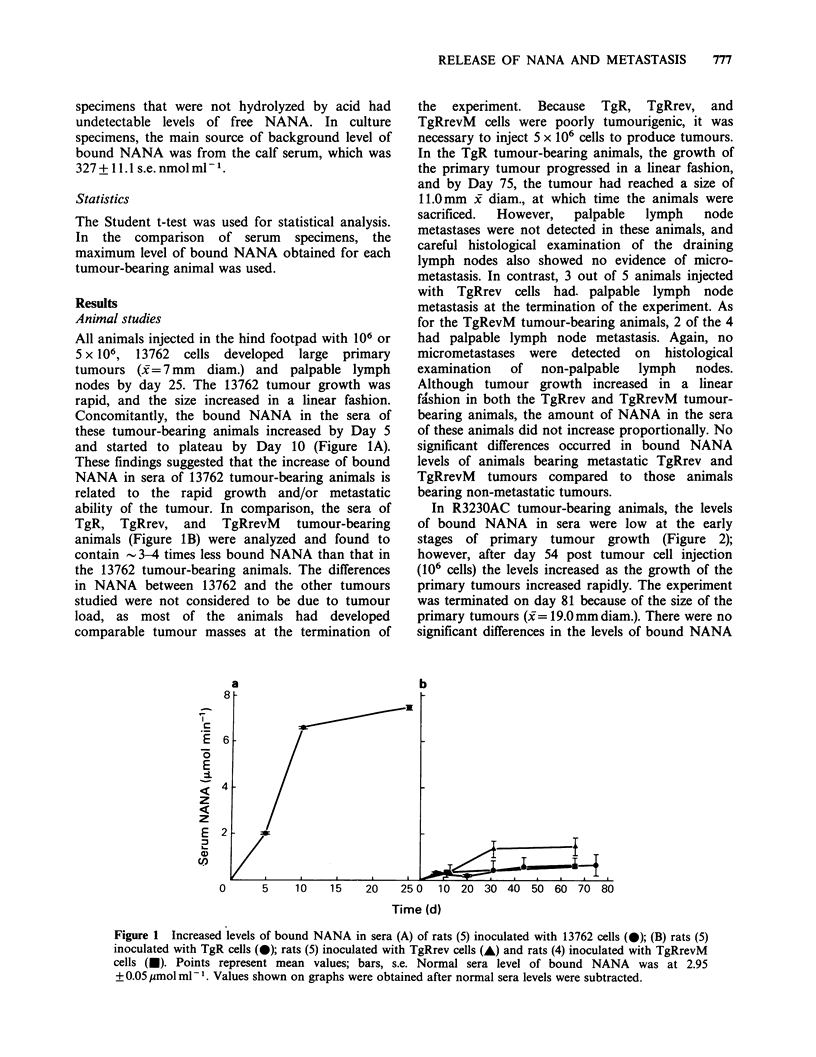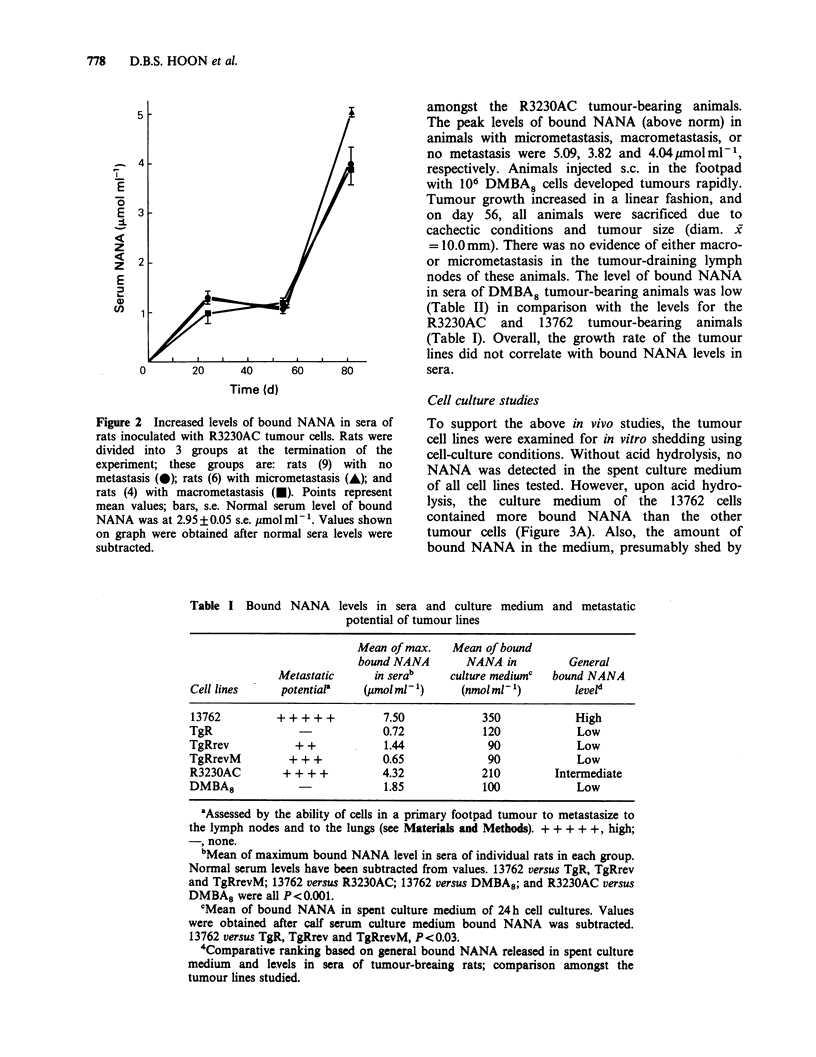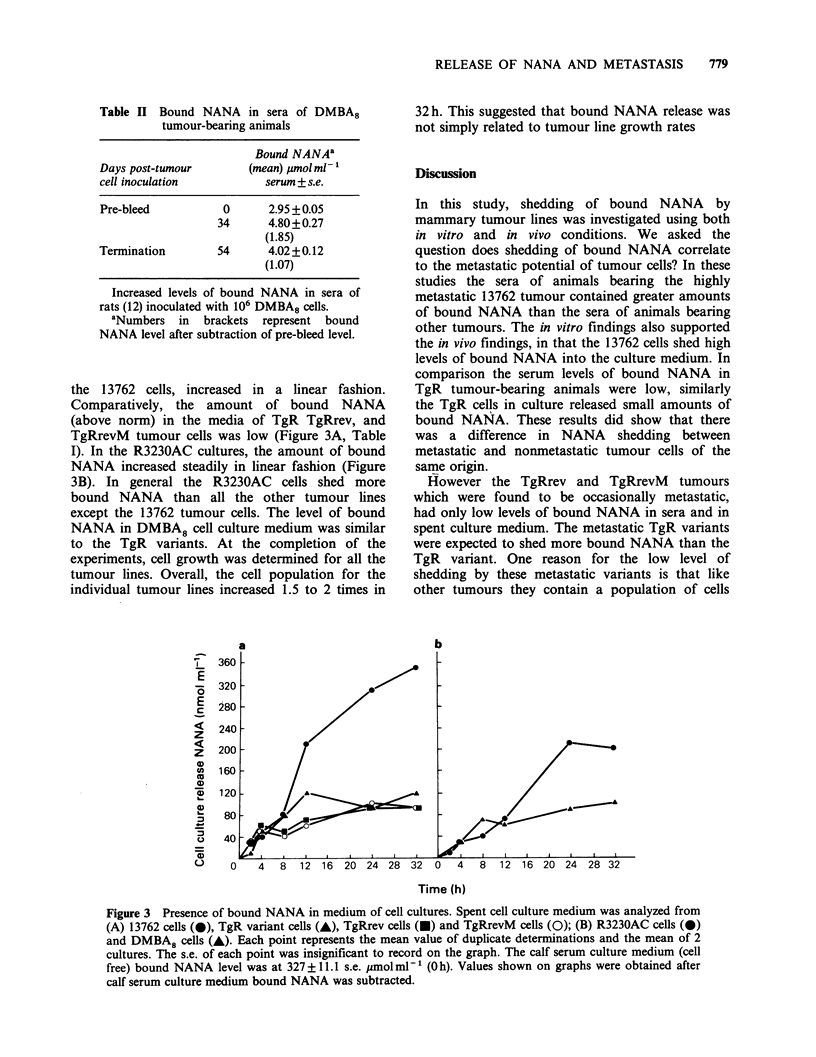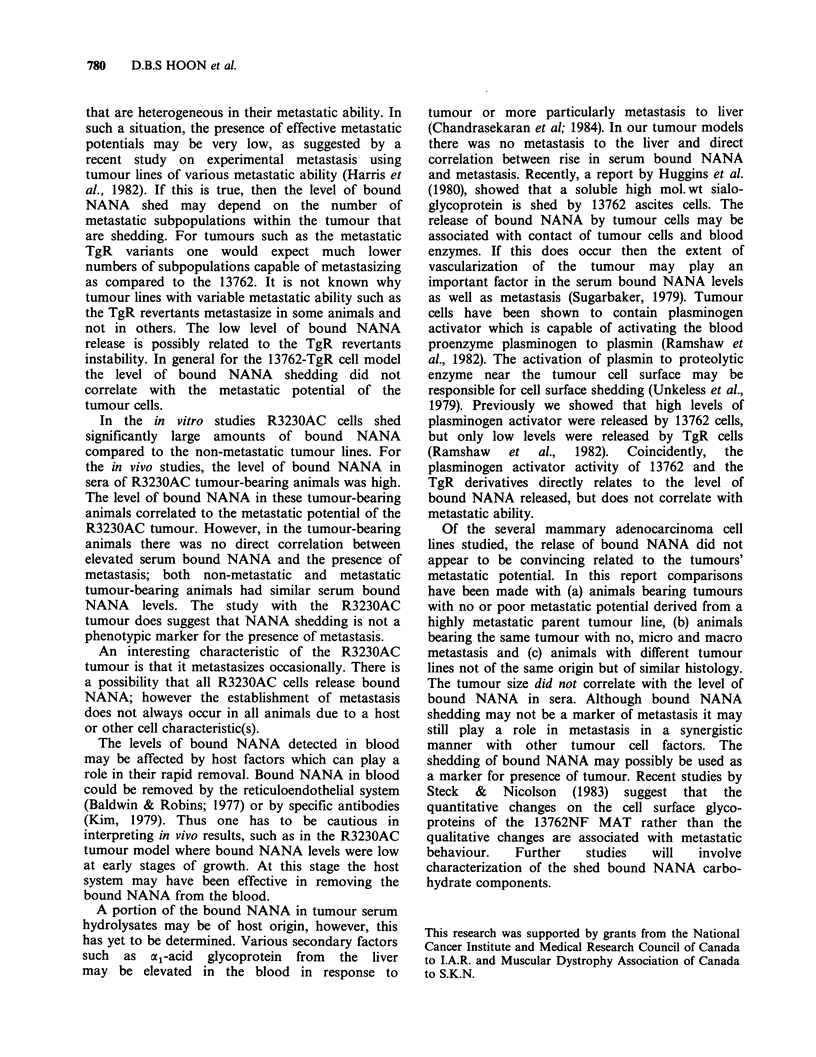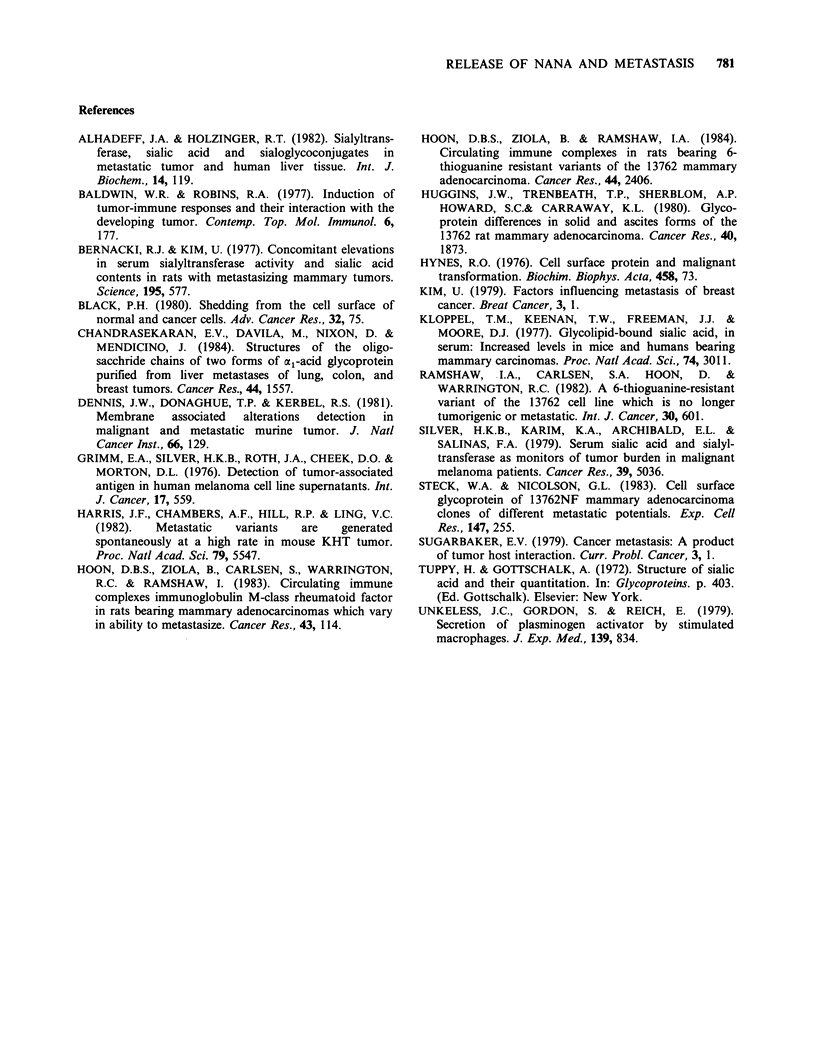# Analysis of mammary tumour cell metastasis and release of bound n-acetylneuraminic acid.

**DOI:** 10.1038/bjc.1985.121

**Published:** 1985-06

**Authors:** D. B. Hoon, S. K. Ng, I. A. Ramshaw

## Abstract

A tumour model consisting of the highly metastatic mammary 13762 parental line, the non-metastatic and 6-thioguanine-resistant (TgR) variant line, and two TgR revertant lines (TgRrev, TgRrevM) that were occasionally metastatic, were used to determine whether the release of N-acetylneuraminic acid (NANA) was related to tumour metastasis. For comparative purposes, the occasionally metastatic R3230AC and the nonmetastatic DMBA8 tumour lines were studied. The NANA was considered to be in bound form, because acid hydrolysis was required to release it for high-pressure liquid chromatographic analyses. Sera of animals bearing the 13762 and R3230AC tumours had high levels of bound NANA. No differences were found in serum NANA levels in animals bearing metastatic or non-metastatic R3230AC tumours. Low levels of bound NANA were found in animals bearing the other tumour lines regardless of whether metastasis occurred or not. The experiments in vitro substantiated the in vivo findings. The phenotypic expression of bound NANA shedding did not correlate well with metastatic potential of the mammary tumour line. Our analyses suggest that this phenotypic marker cannot be used as a reliable indicator of metastasis.


Br. J. Cancer (1985), 51, 775–781

### Analysis of mammary tumour cell metastasis and release of bound n-acetylneuraminic acid

D.B.S. Hoon[1], S.K. Ng[2] & I.A. Ramshaw[3]

[1]*Department of Surgery, Division of Surgical Oncology, 54 –140 CHS, UCLA School of Medicine, Los Angeles, CA 90024, USA;* [2]*Department of Microbiology, Health Science Building, University of Saskatchewan, Saskatoon, Saskatchewan, Canada, S7N 0W0;* [3]*Department of Experimental Pathology, John Curtin School of Medical Research, Australian National University, Canberra City, ACT 2601, Australia.*

**Summary** A tumour model consisting of the highly metastatic mammary 13762 parental line, the non-metastatic and 6-thioguanine-resistant (TgR) variant line, and two TgR revertant lines (TgRrev, TgRrevM) that were occasionally metastatic, were used to determine whether the relase of N-acetylneuraminic acid (NANA) was related to tumour metastasis. For comparative purposes, the occasionally metastatic R3230AC and the nonmetastatic $DMBA_8$ tumour lines were studied. The NANA was considered to be in bound form, because acid hydrolysis was required to release it for high-pressure liquid chromatographic analyses. Sera of animals bearing the 13762 and R3230AC tumours had high levels of bound NANA. No differences were found in serum NANA levels in animals bearing metastatic or non-metastatic R3230AC tumours. Low levels of bound NANA were found in animals bearing the other tumour lines regardless of whether metastasis occurred or not. The experiments *in vitro* substantiated the *in vivo* findings. The phenotypic expression of bound NANA shedding did not correlate well with metastatic potential of the mammary tumour line. Our analyses suggest that this phenotypic marker cannot be used as a reliable indicator of metastasis.

The shedding of cell-surface components may play an important role in the process of metastasis. In both animals and human studies, the level of shedding is considered to be related to metastatic ability (Bernacki & Kim, 1977; Black, 1980; Dennis *et al.*, 1981). The cell components shed can consist of proteases, adhesion molecules, and antigens which may impair the immune reponse (Black, 1980; Hynes, 1976). Tumour cells also seem to have an increased content of sialic acid on cell surface membranes (Alhadeff & Holzinger, 1982; Black, 1980; Huggins *et al.*, 1980) and such bound sialic acid has often been detected in the serum of tumour-bearing hosts and in spent cell-culture medium (Bernacki & Kim, 1977; Grimm *et al.*, 1976; Kloppel *et al.*, 1977; Silver *et al.*, 1979). From these studies it is suggested that shedding of bound sialic acid may relate to metastasis. However, some of the difficulties in validating the shedding hypothesis lies in the fact that (a) in most studies, the *in vitro* and *in vivo* experiments have not been performed simultaneously, (b) the cells employed, though exhibiting varying metastatic potential, generally have different origins and (c) the tumour cell lines are not from the same syngeneic host. In this report we have incorporated the above considerations to determine whether NANA shedding can be used as a phenotypic marker for tumour metastasis.

Correspondence: D.B.S. Hoon
Received 22 October 1984; and in revised form 14 February 1985.

We recently reported the isolation of a 6-thioguanine-resistant cell line from the 13762 mammary adenocarcinoma that is not only much less tumourigenic but also unable to metastasize in normal rats (Ramshaw *et al.*, 1982). Also, we detailed the capacity of other rat mammary adeno-carcinomas to spontaneously metastasize (Hoon *et al.*, 1983). The availability of these tumour cell lines, which vary in their metastatic ability, afforded us the opportunity to study the cell-surface shedding of bound N-acetylneuraminic acid. HPLC analysis was used to quantitate the levels of bound NANA released in specimen hydrolysates. In this report, we show both *in vitro* and *in vivo* that shedding of bound NANA did not correlate to the metastatic potential of the mammary tumour lines.

### Materials and methods

### *Animals and tumour cell lines*

The mammary tumour cell lines used were the 13762, $DMBA_8$, R3230AC, TgR, TgRrev and TgRrevM, all syngeneic to the F344 rat. The tumours were grown and handled as previously reported (Hoon *et al.*, 1983; 1984). The 13762 is a highly metastatic tumour (100%), whereas the R3230AC tumour metastasizes in 45 –100% of the animals. The $DMBA_8$ always grows as a primary tumour but does not metastasize. A 6-thio-guanine(TgR)-resistant variant was obtained from the 13762 cell line, which was found to be poorly

© The Macmillan Press Ltd., 1985

tumourigenic and non-metastatic (Ramshaw et al., 1982). From the TgR variant, two more cell lines were derived, the TgRrev and TgRrevM, which were metastatic and more tumourigenic than the TgR line itself. The differences between the TgRrevM and TgRrev are minor, the former is more metastatic in the lung tumour colony assay (Ramshaw et al., 1982). These metastatic derivatives metastasized in 10 -60% of the animals. The metastatic ability of these tumour lines was assessed by injecting the cells into the hind footpad and assessing lymphatic metastasis in the draining regional lymph nodes at regular intervals (Hoon et al., 1983).

*Serum specimens*

Rats were bled from the tail vein prior to tumour cell injection (pre-bleed sera) and periodically thereafter (Hoon et al., 1983). For each tumour line studied two or more experiments had been carried out.

*NANA assay*

To assay for *in vitro* tumour-cell surface shedding, samples of culture medium were analyzed for NANA. Only cell cultures in log growth phase with >95% viability were used in the assays. Since the 13762, TgR, TgRrev, and TgRrevM cells can grow in suspension, removal of these cells was achieved by gentle agitation of the flask followed by pipetting. The R3230AC and $DMBA_8$ cells adhered firmly to the culture flasks, removal of these cells required washing the cell monolayer several times with 0.02 M EDTA in PBS ($Ca^{2+}$, $Mg^{2+}$ free, pH 7.3) for 5 min at 37°C, followed by gentle agitation of the flask to remove the cells. All cells removed from the culture flasks were then washed three times in RPMI 1640 medium supplemented with 5% calf serum, and seeded at a concentration of $10^6$ cells $ml^{-1}$ in 60 mm tissue culture plates (Falcon, Oxnard, CA). Two plates were seeded for each time point, and then incubated at 37°C in a humidified atmosphere of 5% $CO_2$ and 95% air. At 0, 2, 4, 8, 12, 24 and 32 h intervals, cells wre sampled by first agitating the plate to evenly disperse the cells, followed by the removal of 1.0 ml aliquots. The samples were centrifuged at 1000 *g* for 10 min at room temperature, and the supernatants were stored at −8°C until needed. At the end of each experiment, cells were removed from culture plates and examined for viability by trypan-blue and counted (haemocytometer). At this point, cells were observed to be >85% viable.

Bound NANA both in serum and tissue-culture medium was analyzed by HPLC. The use of HPLC for detection of NANA is reliable, selective, and sensitive (Silver et al., 1979), compared to the colorimetric procedures (Tuppy & Gottschalk, 1972). The NANA was hydrolyzed from its bound form by acid treatment as follows: the sample was diluted 1:20 (v/v) with 0.1 M sulphuric acid, while the culture medium sample was diluted 1:10 with 1 M sulphuric acid. The diluted samples were then heated at 80°C for 1 h. Hydrolysates were then cooled for 15 min in a 20°C water bath, and further diluted 1:1 (v/v) with distilled water. This minimized the interference of the acid peak during HPLC analyses on a model 332 gradient system fitted with model 110A pumps (Beckman, Berkeley, CA) and Aminex HPX-87 cation exchange column (300 × 7.8 mm, Bio-Rad Lab., Richmond, CA). The NANA in the sample was eluted by a mobile phase of 0.006 M sulphuric acid at a flow rate of 0.65 ml $min^{-1}$ and pressure of 7 $+10^6$ $Nm^{-2}$. The eluant was monitored at 206 nm by a model 100–40 spectrophotometer (Hitachi, Tokyo, Japan) fitted with a 5 $\mu$l flow cell and an Altex integrator recorder (Shimadzu Corp., Kyoto, Japan). Samples at 20 $\mu$l were injected into the sampling port via a Hamilton syringe, preset running time for each assay was ~15 min. Each sample was examined at least twice, and interassay variations where <5%.

The amount of NANA in samples was determined by comparing the area of the peak with a standard curve of hydrolyzed NANA (0.15 to 5 $\mu$mol $ml^{-1}$) established by the same HPLC procedure. The lowest concentration that could be detected by this system was 10 nmol $ml^{-1}$. The eulted NANA had a retention time of 7.5 min, while the forerunning acid peak was at 6.0 min; when the tumour serum hydrolysates were assayed, only an acid and a NANA peak were observed. When the NANA standard solution was mixed with tumour serum hydrolysate, there was only one peak at the NANA retention time. On running the tumour serum hydrolysate, culture medium hydrolysate, and standard NANA, in separate runs, the NANA peak retention times were identical. Appropriate concentrations of N-acetyl galactosamine (Sigma, St. Louis, MO) and N-acetyl-mannosamine (Sigma) were incorporated into the samples as internal standards. The retention times did not coincide with the peaks observed with the respective serum hydrolysates; they were at 8.9 and 11.1 min, respectively. Serum hydrolysates could be stored at 4°C for 48 h without any significant loss of NANA concentration. Pre-bleed specimens and the zero time point for culture-medium specimens for each assay were considered as baseline or background levels. The values obtained after this point were expressed in terms of increases over baseline levels. Normal control serum specimens taken at random diurnal periods for sex-age-matched rats were $2.95 \pm 0.05$ s.e. $\mu$mol $ml^{-1}$. Pre-bleed and post tumour injection serum specimens that were not hydrolyzed by acid had undetectable levels of free NANA. In culture specimens, the main source of background level of bound NANA was from the calf serum, which was $327 \pm 11.1$ s.e. nmol ml$^{-1}$.

*Statistics*

The Student t-test was used for statistical analysis. In the comparison of serum specimens, the maximum level of bound NANA obtained for each tumour-bearing animal was used.

### Results

*Animal studies*

All animals injected in the hind footpad with $10^6$ or $5 \times 10^6$, 13762 cells developed large primary tumours ($\bar{x} = 7$ mm diam.) and palpable lymph nodes by day 25. The 13762 tumour growth was rapid, and the size increased in a linear fashion. Concomitantly, the bound NANA in the sera of these tumour-bearing animals increased by Day 5 and started to plateau by Day 10 (Figure 1A). These findings suggested that the increase of bound NANA in sera of 13762 tumour-bearing animals is related to the rapid growth and/or metastatic ability of the tumour. In comparison, the sera of TgR, TgRrev, and TgRrevM tumour-bearing animals (Figure 1B) were analyzed and found to contain ~3–4 times less bound NANA than that in the 13762 tumour-bearing animals. The differences in NANA between 13762 and the other tumours studied were not considered to be due to tumour load, as most of the animals had developed comparable tumour masses at the termination of the experiment. Because TgR, TgRrev, and TgRrevM cells were poorly tumourigenic, it was necessary to inject $5 \times 10^6$ cells to produce tumours. In the TgR tumour-bearing animals, the growth of the primary tumour progressed in a linear fashion, and by Day 75, the tumour had reached a size of 11.0 mm $\bar{x}$ diam., at which time the animals were sacrificed. However, palpable lymph node metastases were not detected in these animals, and careful histological examination of the draining lymph nodes also showed no evidence of micrometastasis. In contrast, 3 out of 5 animals injected with TgRrev cells had palpable lymph node metastasis at the termination of the experiment. As for the TgRevM tumour-bearing animals, 2 of the 4 had palpable lymph node metastasis. Again, no micrometastases were detected on histological examination of non-palpable lymph nodes. Although tumour growth increased in a linear fashion in both the TgRrev and TgRrevM tumour-bearing animals, the amount of NANA in the sera of these animals did not increase proportionally. No significant differences occurred in bound NANA levels of animals bearing metastatic TgRrev and TgRrevM tumours compared to those animals bearing non-metastatic tumours.

In R3230AC tumour-bearing animals, the levels of bound NANA in sera were low at the early stages of primary tumour growth (Figure 2); however, after day 54 post tumour cell injection ($10^6$ cells) the levels increased as the growth of the primary tumours increased rapidly. The experiment was terminated on day 81 because of the size of the primary tumours ($\bar{x} = 19.0$ mm diam.). There were no significant differences in the levels of bound NANA

**Figure 1** Increased levels of bound NANA in sera (A) of rats (5) inoculated with 13762 cells (●); (B) rats (5) inoculated with TgR cells (●); rats (5) inoculated with TgRrev cells (▲) and rats (4) inoculated with TgRrevM cells (■). Points represent mean values; bars, s.e. Normal sera level of bound NANA was at 2.95 $\pm 0.05\,\mu$mol ml$^{-1}$. Values shown on graphs were obtained after normal sera levels were subtracted.

**Figure 2** Increased levels of bound NANA in sera of rats inoculated with R3230AC tumour cells. Rats were divided into 3 groups at the termination of the experiment; these groups are: rats (9) with no metastasis (●); rats (6) with micrometastasis (▲); and rats (4) with macrometastasis (■). Points represent mean values; bars, s.e. Normal serum level of bound NANA was at 2.95±0.05 s.e. $\mu$mol ml$^{-1}$. Values shown on graph were obtained after normal sera levels were subtracted.

amongst the R3230AC tumour-bearing animals. The peak levels of bound NANA (above norm) in animals with micrometastasis, macrometastasis, or no metastasis were 5.09, 3.82 and 4.04 $\mu$mol ml$^{-1}$, respectively. Animals injected s.c. in the footpad with $10^6$ $DMBA_8$ cells developed tumours rapidly. Tumour growth increased in a linear fashion, and on day 56, all animals were sacrificed due to cachectic conditions and tumour size (diam. $\bar{x}$ = 10.0 mm). There was no evidence of either macro- or micrometastasis in the tumour-draining lymph nodes of these animals. The level of bound NANA in sera of $DMBA_8$ tumour-bearing animals was low (Table II) in comparison with the levels for the R3230AC and 13762 tumour-bearing animals (Table I). Overall, the growth rate of the tumour lines did not correlate with bound NANA levels in sera.

*Cell culture studies*

To support the above *in vivo* studies, the tumour cell lines were examined for *in vitro* shedding using cell-culture conditions. Without acid hydrolysis, no NANA was detected in the spent culture medium of all cell lines tested. However, upon acid hydrolysis, the culture medium of the 13762 cells contained more bound NANA than the other tumour cells (Figure 3A). Also, the amount of bound NANA in the medium, presumably shed by

**Table I** Bound NANA levels in sera and culture medium and metastatic potential of tumour lines

| *Cell lines* | *Metastatic potential*[a] | *Mean of max. bound NANA in sera*[b] *($\mu$mol ml$^{-1}$)* | *Mean of bound NANA in culture medium*[c] *(nmol ml$^{-1}$)* | *General bound NANA level*[d] |
|---|---|---|---|---|
| 13762 | + + + + + | 7.50 | 350 | High |
| TgR | — | 0.72 | 120 | Low |
| TgRrev | + + | 1.44 | 90 | Low |
| TgRrevM | + + + | 0.65 | 90 | Low |
| R3230AC | + + + + | 4.32 | 210 | Intermediate |
| $DMBA_8$ | — | 1.85 | 100 | Low |

[a]Assessed by the ability of cells in a primary footpad tumour to metastasize to the lymph nodes and to the lungs (see **Materials and Methods**). + + + + +, high; —, none.

[b]Mean of maximum bound NANA level in sera of individual rats in each group. Normal serum levels have been subtracted from values. 13762 *versus* TgR, TgRrev and TgRrevM; 13762 *versus* R3230AC; 13762 *versus* $DMBA_8$; and R3230AC *versus* $DMBA_8$ were all $P<0.001$.

[c]Mean of bound NANA in spent culture medium of 24 h cell cultures. Values were obtained after calf serum culture medium bound NANA was subtracted. 13762 *versus* TgR, TgRrev and TgRrevM, $P<0.03$.

[d]Comparative ranking based on general bound NANA released in spent culture medium and levels in sera of tumour-breaing rats; comparison amongst the tumour lines studied.

**Table II** Bound NANA in sera of $DMBA_8$ tumour-bearing animals

| *Days post-tumour cell inoculation* | | *Bound NANA[a] (mean) μmol ml$^{-1}$ serum ± s.e.* |
|---|---|---|
| Pre-bleed | 0 | 2.95±0.05 |
| | 34 | 4.80±0.27 (1.85) |
| Termination | 54 | 4.02±0.12 (1.07) |

Increased levels of bound NANA in sera of rats (12) inoculated with $10^6$ $DMBA_8$ cells.

[a]Numbers in brackets represent bound NANA level after subtraction of pre-bleed level.

the 13762 cells, increased in a linear fashion. Comparatively, the amount of bound NANA (above norm) in the media of TgR TgRrev, and TgRrevM tumour cells was low (Figure 3A, Table I). In the R3230AC cultures, the amount of bound NANA increased steadily in linear fashion (Figure 3B). In general the R3230AC cells shed more bound NANA than all the other tumour lines except the 13762 tumour cells. The level of bound NANA in $DMBA_8$ cell culture medium was similar to the TgR variants. At the completion of the experiments, cell growth was determined for all the tumour lines. Overall, the cell population for the individual tumour lines increased 1.5 to 2 times in 32 h. This suggested that bound NANA release was not simply related to tumour line growth rates

### Discussion

In this study, shedding of bound NANA by mammary tumour lines was investigated using both *in vitro* and *in vivo* conditions. We asked the question does shedding of bound NANA correlate to the metastatic potential of tumour cells? In these studies the sera of animals bearing the highly metastatic 13762 tumour contained greater amounts of bound NANA than the sera of animals bearing other tumours. The *in vitro* findings also supported the *in vivo* findings, in that the 13762 cells shed high levels of bound NANA into the culture medium. In comparison the serum levels of bound NANA in TgR tumour-bearing animals were low, similarly the TgR cells in culture released small amounts of bound NANA. These results did show that there was a difference in NANA shedding between metastatic and nonmetastatic tumour cells of the same origin.

However the TgRrev and TgRrevM tumours which were found to be occasionally metastatic, had only low levels of bound NANA in sera and in spent culture medium. The metastatic TgR variants were expected to shed more bound NANA than the TgR variant. One reason for the low level of shedding by these metastatic variants is that like other tumours they contain a population of cells

**Figure 3** Presence of bound NANA in medium of cell cultures. Spent cell culture medium was analyzed from (A) 13762 cells (●), TgR variant cells (▲), TgRrev cells (■) and TgRrevM cells (○); (B) R3230AC cells (●) and $DMBA_8$ cells (▲). Each point represents the mean value of duplicate determinations and the mean of 2 cultures. The s.e. of each point was insignificant to record on the graph. The calf serum culture medium (cell free) bound NANA level was at 327±11.1 s.e. μmol ml$^{-1}$ (0 h). Values shown on graphs were obtained after calf serum culture medium bound NANA was subtracted.

that are heterogeneous in their metastatic ability. In such a situation, the presence of effective metastatic potentials may be very low, as suggested by a recent study on experimental metastasis using tumour lines of various metastatic ability (Harris *et al.,* 1982). If this is true, then the level of bound NANA shed may depend on the number of metastatic subpopulations within the tumour that are shedding. For tumours such as the metastatic TgR variants one would expect much lower numbers of subpopulations capable of metastasizing as compared to the 13762. It is not known why tumour lines with variable metastatic ability such as the TgR revertants metastasize in some animals and not in others. The low level of bound NANA release is possibly related to the TgR revertants instability. In general for the 13762-TgR cell model the level of bound NANA shedding did not correlate with the metastatic potential of the tumour cells.

In the *in vitro* studies R3230AC cells shed significantly large amounts of bound NANA compared to the non-metastatic tumour lines. For the *in vivo* studies, the level of bound NANA in sera of R3230AC tumour-bearing animals was high. The level of bound NANA in these tumour-bearing animals correlated to the metastatic potential of the R3230AC tumour. However, in the tumour-bearing animals there was no direct correlation between elevated serum bound NANA and the presence of metastasis; both non-metastatic and metastatic tumour-bearing animals had similar serum bound NANA levels. The study with the R3230AC tumour does suggest that NANA shedding is not a phenotypic marker for the presence of metastasis.

An interesting characteristic of the R3230AC tumour is that it metastasizes occasionally. There is a possibility that all R3230AC cells release bound NANA; however the establishment of metastasis does not always occur in all animals due to a host or other cell characteristic(s).

The levels of bound NANA detected in blood may be affected by host factors which can play a role in their rapid removal. Bound NANA in blood could be removed by the reticuloendothelial system (Baldwin & Robins; 1977) or by specific antibodies (Kim, 1979). Thus one has to be cautious in interpreting *in vivo* results, such as in the R3230AC tumour model where bound NANA levels were low at early stages of growth. At this stage the host system may have been effective in removing the bound NANA from the blood.

A portion of the bound NANA in tumour serum hydrolysates may be of host origin, however, this has yet to be determined. Various secondary factors such as $\alpha_1$-acid glycoprotein from the liver may be elevated in the blood in response to tumour or more particularly metastasis to liver (Chandrasekaran *et al;* 1984). In our tumour models there was no metastasis to the liver and direct correlation between rise in serum bound NANA and metastasis. Recently, a report by Huggins *et al.* (1980), showed that a soluble high mol. wt sialo-glycoprotein is shed by 13762 ascites cells. The release of bound NANA by tumour cells may be associated with contact of tumour cells and blood enzymes. If this does occur then the extent of vascularization of the tumour may play an important factor in the serum bound NANA levels as well as metastasis (Sugarbaker, 1979). Tumour cells have been shown to contain plasminogen activator which is capable of activating the blood proenzyme plasminogen to plasmin (Ramshaw *et al.,* 1982). The activation of plasmin to proteolytic enzyme near the tumour cell surface may be responsible for cell surface shedding (Unkeless *et al.,* 1979). Previously we showed that high levels of plasminogen activator were released by 13762 cells, but only low levels were released by TgR cells (Ramshaw *et al.,* 1982). Coincidently, the plasminogen activator activity of 13762 and the TgR derivatives directly relates to the level of bound NANA released, but does not correlate with metastatic ability.

Of the several mammary adenocarcinoma cell lines studied, the relase of bound NANA did not appear to be convincing related to the tumours' metastatic potential. In this report comparisons have been made with (a) animals bearing tumours with no or poor metastatic potential derived from a highly metastatic parent tumour line, (b) animals bearing the same tumour with no, micro and macro metastasis and (c) animals with different tumour lines not of the same origin but of similar histology. The tumour size *did not* correlate with the level of bound NANA in sera. Although bound NANA shedding may not be a marker of metastasis it may still play a role in metastasis in a synergistic manner with other tumour cell factors. The shedding of bound NANA may possibly be used as a marker for presence of tumour. Recent studies by Steck & Nicolson (1983) suggest that the quantitative changes on the cell surface glyco-proteins of the 13762NF MAT rather than the qualitative changes are associated with metastatic behaviour. Further studies will involve characterization of the shed bound NANA carbo-hydrate components.

This research was supported by grants from the National Cancer Institute and Medical Research Council of Canada to I.A.R. and Muscular Dystrophy Association of Canada to S.K.N.

**References**

ALHADEFF, J.A. & HOLZINGER, R.T. (1982). Sialyltransferase, sialic acid and sialoglycoconjugates in metastatic tumor and human liver tissue. *Int. J. Biochem.*, **14**, 119.

BALDWIN, W.R. & ROBINS, R.A. (1977). Induction of tumor-immune responses and their interaction with the developing tumor. *Contemp. Top. Mol. Immunol.* **6**, 177.

BERNACKI, R.J. & KIM, U. (1977). Concomitant elevations in serum sialyltransferase activity and sialic acid contents in rats with metastasizing mammary tumors. *Science*, **195**, 577.

BLACK, P.H. (1980). Shedding from the cell surface of normal and cancer cells. *Adv. Cancer Res.*, **32**, 75.

CHANDRASEKARAN, E.V., DAVILA, M., NIXON, D. & MENDICINO, J. (1984). Structures of the oligosacchride chains of two forms of $\alpha_1$-acid glycoprotein purified from liver metastases of lung, colon, and breast tumors. *Cancer Res.*, **44**, 1557.

DENNIS, J.W., DONAGHUE, T.P. & KERBEL, R.S. (1981). Membrane associated alterations detection in malignant and metastatic murine tumor. *J. Natl Cancer Inst.*, **66**, 129.

GRIMM, E.A., SILVER, H.K.B., ROTH, J.A., CHEEK, D.O. & MORTON, D.L. (1976). Detection of tumor-associated antigen in human melanoma cell line supernatants. *Int. J. Cancer*, **17**, 559.

HARRIS, J.F., CHAMBERS, A.F., HILL, R.P. & LING, V.C. (1982). Metastatic variants are generated spontaneously at a high rate in mouse KHT tumor. *Proc. Natl Acad. Sci.* **79**, 5547.

HOON, D.B.S., ZIOLA, B., CARLSEN, S., WARRINGTON, R.C. & RAMSHAW, I. (1983). Circulating immune complexes immunoglobulin M-class rheumatoid factor in rats bearing mammary adenocarcinomas which vary in ability to metastasize. *Cancer Res.*, **43**, 114.

HOON, D.B.S., ZIOLA, B. & RAMSHAW, I.A. (1984). Circulating immune complexes in rats bearing 6-thioguanine resistant variants of the 13762 mammary adenocarcinoma. *Cancer Res.*, **44**, 2406.

HUGGINS, J.W., TRENBEATH, T.P., SHERBLOM, A.P. HOWARD, S.C.& CARRAWAY, K.L. (1980). Glycoprotein differences in solid and ascites forms of the 13762 rat mammary adenocarcinoma. *Cancer Res.*, **40**, 1873.

HYNES, R.O. (1976). Cell surface protein and malignant transformation. *Biochim. Biophys. Acta*, **458**, 73.

KIM, U. (1979). Factors influencing metastasis of breast cancer. *Breat Cancer*, **3**, 1.

KLOPPEL, T.M., KEENAN, T.W., FREEMAN, J.J. & MOORE, D.J. (1977). Glycolipid-bound sialic acid, in serum: Increased levels in mice and humans bearing mammary carcinomas. *Proc. Natl Acad. Sci.*, **74**, 3011.

RAMSHAW, I.A., CARLSEN, S.A. HOON, D. & WARRINGTON, R.C. (1982). A 6-thioguanine-resistant variant of the 13762 cell line which is no longer tumorigenic or metastatic. *Int. J. Cancer*, **30**, 601.

SILVER, H.K.B., KARIM, K.A., ARCHIBALD, E.L. & SALINAS, F.A. (1979). Serum sialic acid and sialyltransferase as monitors of tumor burden in malignant melanoma patients. *Cancer Res.*, **39**, 5036.

STECK, W.A. & NICOLSON, G.L. (1983). Cell surface glycoprotein of 13762NF mammary adenocarcinoma clones of different metastatic potentials. *Exp. Cell Res.*, **147**, 255.

SUGARBAKER, E.V. (1979). Cancer metastasis: A product of tumor host interaction. *Curr. Probl. Cancer*, **3**, 1.

TUPPY, H. & GOTTSCHALK, A. (1972). Structure of sialic acid and their quantitation. In: *Glycoproteins*. p. 403. (Ed. Gottschalk). Elsevier: New York.

UNKELESS, J.C., GORDON, S. & REICH, E. (1979). Secretion of plasminogen activator by stimulated macrophages. *J. Exp. Med.*, **139**, 834.